# Examining the effectiveness of promotional nudges increasing plant-based food choices in a post-secondary education dining hall: a pilot study

**DOI:** 10.1017/S1368980024001915

**Published:** 2024-10-10

**Authors:** Jennifer Joy Anderson, Andy Bains, Julie Stachiw, Alexandra J Heidl, Tamara Paetsch, Tamara R Cohen

**Affiliations:** 1 School of Population and Public Health, Faculty of Medicine, The University of British Columbia, Vancouver, BC, Canada; 2 Faculty of Land and Food Systems, The University of British Columbia, Vancouver, BC V6T 1Z4, Canada; 3 Nutrition and Wellbeing, The University of British Columbia, Okanagan Campus, Kelowna, BC, Canada; 4 School of Human Nutrition, McGill University, Ste. Anne de Bellevue, QC, Canada; 5 Nutrition and Wellbeing, The University of British Columbia, Vancouver, BC, Canada

**Keywords:** Nudge theory, Nudging, Plant-based, Post-secondary, Canada’s Food Guide

## Abstract

**Objective:**

To evaluate nudge strategies that increase the consumption of plant-based foods, defined as vegetarian or vegan food items, compared with meat-based options in post-secondary dining hall settings.

**Design:**

A pilot study.

**Setting:**

This study took place in the University of British Columbia Vancouver Campus’s Gather Dining Hall (GDH) over a 6-week intervention period and two control periods. The intervention incorporated several nudges (proportion increases, item placement, taste-focused labelling, Chef’s featured special verbal prompts, social media and promotional posters) into the menu and dining hall area with the goal of increasing the purchases of plant-based items. Sales data from meals that were purchased during the intervention period were compared with sales data from the two control periods.

**Participants:**

Students and staff who purchased meals in the GDH.

**Results:**

The proportion of plant-based items sold significantly increased during the intervention period (56·7 %; *P* < 0·01) compared with the last 6 weeks of term one (53·6 %) and the first 6 weeks of term two (53·4 %). The proportion of plant-based ‘main’ menu items was significantly higher in the intervention period (46·4; *P* < 0·01) when compared with the last 6 weeks of term one (40·9 %) and the first 6 weeks of term two (41·7 %).

**Conclusions:**

The combination of nudges was effective at significantly increasing the selection of plant-based options over meat-based options in a post-secondary dining hall setting.

For young adults, attending post-secondary education often results in increased consumption of calories, sugar, fat and Na, and a decreased consumption of fruit and vegetables^(1–7)^. With intentional design, university dining halls can be a place to influence the dietary choices of young adults towards healthier options and provide unique opportunities to develop food literacy skills^(5–7)^.

The promotion of plant-based foods as a way to encourage healthier dietary choices is increasing in popularity within university dining halls^(8–11)^. This approach aligns with national dietary guidance from Canada’s Dietary Guidelines, which informs Canada’s Food Guide^(12)^. Several studies have shown decreased all-cause mortality when processed and red meats are replaced with plant-based protein sources^(13–15)^. However, changing food behaviours towards more plant-based consumption can be challenging. Nudging, a term derived from the Nudge Theory by Thaler and Sunstein,^(16)^ utilises techniques called ‘choice architecture’ that apply positive and gentle persuasion to encourage behaviour change^(10,11,17–21)^. In Nudge Theory, the individual’s freedom of choice is retained, while the environment is changed to influence easier decision-making and guide the individual to make healthier choices^(16,22)^. Nudge strategies implemented in post-secondary institutions have been shown to increase fruit and vegetable consumption^(23)^ and overall healthier food selection by 38 %^(11)^ while decreasing meat-based meal sales by 10 %-points and increasing plant-based meal sales by 6 %-points^(18)^.

It remains unclear how a combination of nudge strategies would improve plant-based choices over meat-based choices in a Canadian post-secondary context. Therefore, the primary objective of this study was to determine the effects of a combination of nudge-based interventions on increasing the consumption of plant-based food options over meat-based food options in a Canadian post-secondary dining hall setting.

## Methods

### Setting and participants

This study took place in a first-year residence dining hall at the University of British Columbia (Vancouver, British Columbia, Canada) at three different time points: the last 6 weeks of term one (control period 1: October–December 2021), the first 6 weeks of term two (control period 2: January–mid-February 2022) and the last 6 weeks of term two (intervention period: End-February–April 2022). Two control periods at different time points (i.e. start of semester, end of semester) were selected to account for potential differences in diner purchasing habits that may arise throughout the year.

For this study, meals that did not contain animal flesh (vegetarian and vegan meal items) were considered to be plant-based, while any items that included animal flesh were considered to be meat-based.

The dining hall used a declining balance meal plan model^(24)^. Diners not on the meal plan were able to purchase meals at a slightly higher price. The dining hall featured different food stations, with a variety of plant-based and meat-based meal options available. Plant-based options were similarly priced or less expensive than the meat-based options.

### Nudging interventions

A summary of the nudging interventions can be found in Table 1

**Table 1.** Summary of nudging intervention

Nudging Intervention	Specifics
Proportion increases	The proportion of plant-based mains was increased at two stations to be equal to the proportion of meat-based mains. No new items were introduced, but the menu rotation was adjusted, and chicken tenders were removed from the menu so that, at minimum, 50 % of the items served at each station were plant-based during the intervention period
Item placement	Plant-based items were moved and listed as the first item on dining hall and online menus
Taste-focused labelling	Words that highlighted the animal-free aspects of a dish were removed and were replaced with taste-focused labelling using the Edgy Veggies Toolkit offered by Stanford’s SPARQ tools^(30)^. This approach utilises descriptions of specific flavours, ingredients and preparation methods to elevate diners’ expectations of a positive taste experience. For example, dishes such as ‘Marinated tofu’ and ‘Pasta salad’ were renamed to ‘Fresh avocado and ponzu marinated tofu salad’ and ‘Tuscan sundried tomato rotini salad’ respectively.
Chef’s Pick	Working closely with dining hall personnel (dietitians, sous chefs), plant-based items that met Canada’s Food Guide Friendly principles^(31)^ were selected to be promoted as a ‘Chef’s Pick’. A single menu item from each meal (breakfast, lunch, dinner) was highlighted each week as the ‘Chef’s Pick’. These Chef’s Pick items were incorporated into posters featuring Health Canada’s Food Guide Friendly branding (Fig. 1). The Chef’s Pick was also promoted by the university’s dining hall marketing team. Social media promotion for the Chef’s Pick occurred via the dining hall’s Food Service Instagram pages. One social media post per week (six over the intervention period) was released in collaboration with the university’s marketing teams to promote a Chef’s Pick. The posts featured Health Canada branding using vibrant photos of the Chef’s Pick dish. The design/timing of the social media post was decided by the marketing team.
Verbal prompts	Dining hall staff were provided with sample prompts to promote the Chef’s Pick items by speaking about their flavourful components to students during meal selection. Examples of prompting sentences included: • Have you had a chance to try ____? It’s our chef’s choice. • We use ___ in our chef’s choice. Have you tried it yet?
Health Canada Promotional materials	Promotional materials designed by Health Canada^(31)^ (i.e. digital and paper posters) were used to promote plant-based foods. Posters promoting specific food items were placed next to the item being promoted (i.e. posters promoting fruit and vegetable selection were placed near fruit baskets). Digital posters were used in the weekly social media posts. Food Guide Friendly posters were placed in entryways and at serving stations at the staff’s discretion; these materials were rotated over the 6 weeks

**Fig. 1:**
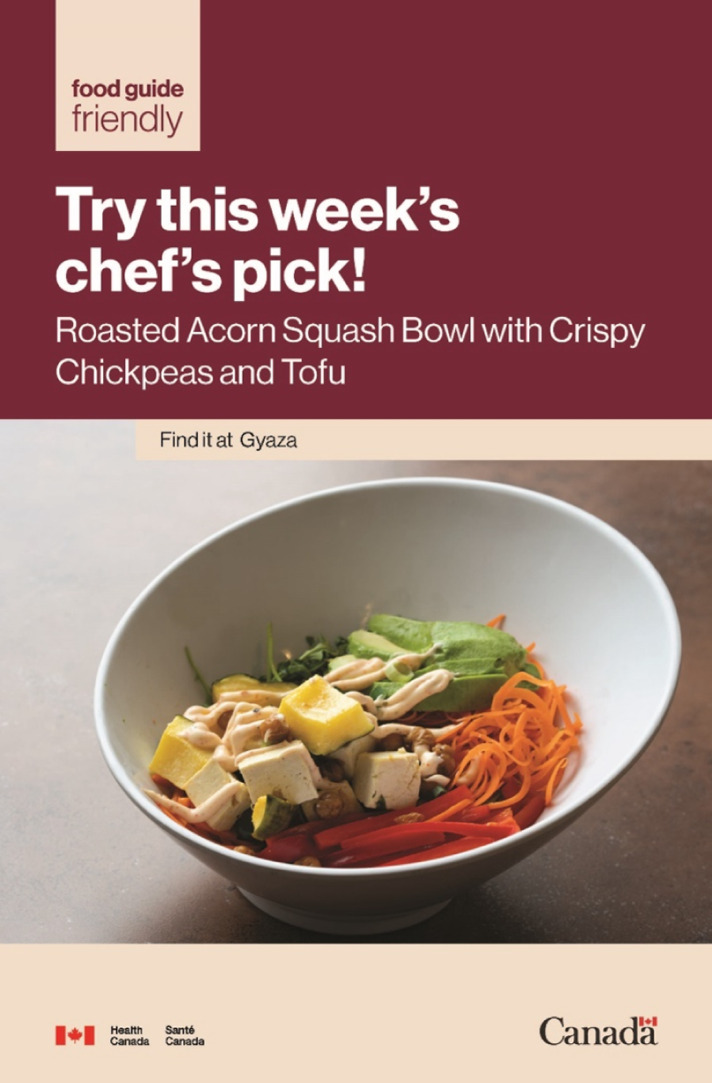
Chef’s pick poster featuring Health Canada’s Food Guide friendly branding

### Statistical analyses

Descriptive statistics (percentages) were used to summarise the proportion of plant-based meals offered during the intervention period. The menu was split into ‘mains’ and ‘sides’ (e.g. fries, side salads, breads/roles, sides of vegetables, etc.) based on how they were coded in the dining hall point-of-sale system or how they were listed on the online dining hall menu. This division was made as sides were mostly plant-based and there were concerns about this artificially inflating the proportion of plant-based items available.

*Grab and Go* items, salad bar items, fruit, drinks, baked goods, breakfast cereals and desserts were all excluded from this analysis. Meals served during the intervention and control periods were separated between meat-based and plant-based categories and then weighted depending on their recurrence within the 6-week period. The recurrence of food items was determined by analysing the number of days, weeks and mealtimes (i.e. breakfast, lunch or dinner) they were served.

Descriptive statistics (mean, percentage, frequencies) were used to summarise point of sale data extracted from the dining hall database for three time periods (control period 1, control period 2 and the intervention period). Chi-square tests were conducted to assess differences between the intervention and control periods. Data were analysed using R Studio (version 4·0·2), with a significance level set at *P* < 0·05.

## Results

### Plant-based offerings during the intervention period

During the intervention period, 60·0 % of all food items being offered were plant-based compared to items containing meat. When looking at side and main subcategories, 85·7 % of side menu offerings were plant-based, whereas 61·9 % of main menu offerings were plant-based.

### Food sales

During the intervention period, the proportion of plant-based meal sales significantly increased: 56·7 % of the sales were plant-based during the intervention period compared with 53·6 % and 53·4 % of sales during control periods 1 and 2 (*P* < 0·01) (Table 2). Of these items, the proportion of plant-based main sales significantly increased across term periods with sales at 46·4 % during the intervention period compared with 40·9 % and 41·7 % in control period 1 and 2, respectively (*P* < 0·01). In contrast, the proportion of plant-based side item sales significantly decreased during the intervention period, with 76·3 % of sales classified as plant-based items during the intervention period, down from 79·2 % and 77·6 % in control period 1 and 2 (*P* < 0·01).

**Table 2.** Proportion of meal sales at gather dining hall that are plant-based in intervention period compared with two comparison periods

	Term one Oct 27–Dec 8 2021		Term two Jan 10–Feb 18 2022		Intervention Feb 28–April 8 2022		
	*n*	%	*n*	%	*n*	%	*P*-value
Total items sold (unit items)							
	55 966		45 023		46 516		
All menu items (unit items (%))							< 0·01
Plant-based	29 997·	53·6	24 080	53·4	26 368	56·7	
Meat-based	25 969·	46·4	20 963	46·6	20 148	43·3	
Menu items: mains (unit items (%))							< 0·01
Plant-based	15 269·	40·9	12 602	41·7	14 173	46·4	
Meat-based	22 106·	59·1	17 652	58·3	16 366	53·5	
Menu items: sides (unit items (%))							< 0·01
Plant-based	14 728·	79·2	11 458	77·6	12 195	76·3	
Meat-based	3863·	20·8	3311	22·4	3782	23·7	

Frequency counts (*n*) and percentages (%) are shown as appropriate.

Chi-squared test is used for all categorical *P*-value calculations.

## Discussion

Overall, the implementation of multiple nudge strategies to encourage plant-based meals within a post-secondary dining hall setting resulted in an increase in the purchase of plant-based items through the combination of different strategies. While the influence of each individual strategy on increasing plant-based meals is unknown, others have shown that when these nudge strategies are individually implemented in post-secondary cafeterias, positive and significant results are seen^(8,17,19,25,26)^.

Cost and ease of implementation are important considerations when selecting nudge-based strategies. Numerous nudges have been shown to alter dietary decisions^(10,27)^; however, dining hall staff in this study advised that they were not feasible to implement or maintain. This is similar to others who suggest that interventions become less feasible or sustainable when they require additional staff, incorporate activities (e.g. supplying free samples), create default menus or create wait times for less healthy meals^(10,27)^.

Comparable increases in plant-based meal purchases have been seen in other studies when the quantity of plant-based items offered was increased^(21)^ or when some meat-based items were removed from the menu ‘forcing’ students to choose other options^(9)^. Both strategies were implemented within the dining hall, with stations increasing plant-based meal offerings and removing chicken tenders from the menu, a decision made by the dining hall chef in hopes that by removing this popular main dish, students would try other options. Further, menus were restructured to position plant-based items at the top during the intervention period. Previously, this strategy has been shown to increase sales by six percentage points, with the increase being attributed to ‘order effects’ where items lower on the menu are less likely to be selected due to reader’s fatigue^(18)^.

An interesting finding in this study was that we saw an increase in the proportion of sales for meat-based side dish items during the intervention period. We speculate that students were seeking more variety in their second semester or had become more restrictive with their spending due to the declining balance model. Nonetheless, to encourage plant-based over meat-based meal purchases, many of the plant-based ‘mains’ were renamed during the intervention period to incorporate taste-focused labelling. It was thought that this change in labelling, which was more descriptive of the meal, would appeal more to the consumer^(11)^.

In this study, the Chef’s Pick promotions implemented were similar to strategies used by Broers et al.^(19)^ which demonstrated 1·70 increased odds of selecting the ‘chef’s suggestion’ meal. Previous studies also found the use of verbal prompts by cafeteria staff and promotional materials helped nudge diners towards more healthful meals in post-secondary dining halls^(8,17,25,26)^. Although not a traditional ‘nudge’ method, this study also incorporated social media campaigns to help promote the Chef’s Pick, a strategy suggested to influence the ‘social norms’ of the students^(20)^. It is possible that this strategy may have contributed to our results.

Other promotional ‘nudge’ strategies, such as placing health-based promotional materials next to items have been found to make diners ‘more aware of healthy food choices’ and encourage changes in eating habits^(17,26)^. Given previous research, it is reasonable to assume that the strategic placement of Health Canada’s Food Guide-Friendly promotional materials, implemented during this study, was one of the factors that encouraged the gravitation towards more plant-based foods.

### Strengths

Sustainable and feasible nudges were a critical aspect of this study. The research team worked closely with dining hall staff to design and adapt nudges as needed for implementation, which is unique, as other studies have nudges implemented only by the researchers^(11)^. Further, our interventions were simple to implement and easy to maintain for the staff. As there were no changes to the menu and no new recipes or meals that needed to be developed, the chosen interventions did not significantly change the operations of the dining hall. As dining hall staff were responsible for the placement and rotation of the promotion materials and ensuring the staff used the verbal prompts, this limited the researchers influence on the nudge strategies yet allowed for a better reflection of a real-word dining hall operation and made the results more generalisable to a non-study setting.

Finally, we used two control periods to account for the potential changes in dietary patterns and spending habits throughout the year. A study by Wansink et al.^(27)^ found an increase of 0·4 % per week in unhealthier snack food selections, with a sharp rise of 8 % during the last 2 weeks of the semester. To address this, the last 6 weeks of term one was selected as a control period to account for any end-of-semester effects on diet and spending habits^(27)^. During the second semester, financial stress, such as having a low balance on their meal plan,^(28,29)^ could also affect students’ dietary choices. Therefore, the first 6 weeks of term two were selected as a second control period to account for any second semester effects on diet or spending habits.

### Limitations

As the dining hall in this study serves largely first-year students, these results may not be generalisable to dining halls that serve senior students. Due to the duration of this study, the study design did not allow us to take a ‘segmented approach’ to understanding which nudge strategy was most effective. Additionally, this study occurred during the COVID-19 pandemic when the transition from online to on-campus classes may have impacted how many diners purchased meals within the residential dining halls. The mental and financial well-being of the students during the pandemic may also have altered the dietary behaviours, which was not accounted for in this study. Another limitation of this study is the Point of Sales (POS) system used for sales transactions in the dining hall. As the POS system only tracks sales of items sold, it was not possible to report on any trends in proportion of meal types purchased by each student/dining hall patron.

Finally, the use of vegetarian and vegan foods as an indicator for plant-based foods is limited. While this enables a clear distinction between plant-based and non-plant-based foods, it cannot distinguish the health properties of a food item; therefore, an item considered as plant-based did not necessarily result in a healthier selection than a meat-based alternative, particularly when simulated meat products were used.

## Conclusion

This project capitalised upon the plant-based options that were already available at a post-secondary dining hall setting and actively promoted them using various nudging techniques. Importantly, the findings from this study contribute to the literature on using nudges in post-secondary dining halls. Future studies should explore combinations of behavioural nudges through a step-wise manner. Similarly, the efficacy of behavioural nudges emphasising the environmental impact of dietary choices, as opposed to those focusing on health-related impacts, are worthy of investigation. Finally, while nudges are meant to be subtle, it would be worth investigating the perception of students’ own choices made during exposure to the nudges and to investigate if there are other factors driving student food choices.
